# EFFECT OF COVID-19 PANDEMIC ON A BODY MASS INDEX OF OLDER AMERICANS: A REPEATED CROSS-SECTIONAL STUDY

**DOI:** 10.1093/geroni/igad104.2263

**Published:** 2023-12-21

**Authors:** Krishna Sapkota, J Scott Brown, Jennifer Kinney

**Affiliations:** Miami University, Oxford, Ohio, Oxford, Ohio, United States; Miami University, Oxford, Ohio, United States; Miami University, Oxford, Ohio, United States

## Abstract

**Introduction:**

The COVID-19 pandemic resulted in greater social and physical isolation, potentially leading to changes in eating, physical activity, and health behaviors, which may increase BMI. This study explored whether body mass index (BMI) among middle-aged and older Americans has been affected by the pandemic.

**Methods:**

This study uses data from the Behavioral Risk Factor Surveillance System to assess the effects of COVID-19 on BMI changes among middle-aged (50-64 years) and older (65 years and above) Americans. Changes in BMI over time are measured for 2019 and 2021. Data from 2013, 2015, and 2017 are used to note the trend of BMI, and sex and racial differences in BMI trajectories are explored.

**Results:**

Initial results show that the BMI of older adults increases over time but does so at a decreasing rate (2013 to 2105 BMI difference = 0.19, 0.16 from 2015 to 2017, 0.13 from 2017 to 2019, and 0.09 from 2019 to 2021). On the other hand, among middle-aged adults, BMI significantly increased over time and accelerated during COVID-19. The difference in BMI was 0.20 from 2013 to 2015, 0.16 from 2015 to 2017, 0.22 from 2017 to 2019, and 0.28 from 2019 to 2021. BMI increases consistently over the period for middle-aged men and older adult women, whereas BMI growth accelerates for middle-aged women and decelerates for older adult men. Similarly, racial differences are noted in the BMI changes of middle-aged and older adults.

**Conclusion:**

The COVID-19 pandemic accelerated the accumulation of body mass among middle-aged Americans.

